# HIV Infection Enhances TRAIL-Induced Cell Death in Macrophage by Down-Regulating Decoy Receptor Expression and Generation of Reactive Oxygen Species

**DOI:** 10.1371/journal.pone.0018291

**Published:** 2011-04-05

**Authors:** Dan-Ming Zhu, Juan Shi, Shilian Liu, Yanxin Liu, Dexian Zheng

**Affiliations:** National Laboratory of Medical Molecular Biology, Institute of Basic Medical Sciences, Chinese Academy of Medical Sciences and Peking Union Medical College, Beijing, China; Beijing Institute of Infectious Diseases, China

## Abstract

**Background:**

Tumor necrosis factor-related apoptosis-inducing ligand (TRAIL) could induce apoptosis of HIV-1-infected monocyte-derived macrophage (MDM), but the molecular mechanisms are not well understood.

**Methodology/Principal Findings:**

By using an HIV-1 Env-pseudotyped virus (HIV-1 PV)-infected MDM cell model we demonstrate that HIV-1 PV infection down-regulates the expression of TRAIL decoy receptor 1 (DcR1) and 2 (DcR2), and cellular FLICE-inhibitory protein (c-FLIP), but dose not affect the expression of death receptor 4 and 5 (DR4, DR5), and Bcl-2 family members in MDM cells. Furthermore, recombinant soluble TRAIL and an agonistic anti-DR5 antibody, AD5-10, treatment stimulates reactive oxygen species (ROS) generation and JNK phosphorylation.

**Conclusions/Significance:**

HIV infection facilitates TRIAL-induced cell death in MDM by down-regulating the expression of TRAIL decoy receptors and intracellular c-FLIP. Meanwhile, the agonistic anti-DR5 antibody, AD5-10, induces apoptosis synergistically with TRAIL in HIV-1-infected cells. ROS generation and JNK phosphorylation are involved in this process. These findings potentiate clinical usage of the combination of TRAIL and AD5-10 in eradication of HIV-infected macrophage and AIDS.

## Introduction

HIV infection of macrophages is a critically important component of viral pathogenesis and progression to AIDS. Macrophage contributes an important cellular target for R5-tropic strains of HIV-1 and could disseminates the virus to diverse tissues and organs [1]. HIV-1-infected macrophage is considered as the source not only of viral proteins but also of many inflammatory cytokines, which lead to recruitment of additional susceptible T cells to the primary infection site and contribute to that more cells are infected [1], [2]. Furthermore, macrophage dysfunction as well as induction of immune response is responsible for HIV-associated disorder and AIDS development [3], [4], [5], [6]. It is reported that several aspects of virus-host interaction are unique to macrophage in contrast to T cell, which enables HIV-infected macrophage to be hardly recognized and eliminated by host immune system. Thus cells of macrophage lineage provide an important viral reservoir in vivo and play critical roles in early-stage viral transmission and viral persistence [7], [8]. Therefore, development of therapeutic strategies or agents targeting HIV-infected macrophage is urgently needed.

Tumor necrosis factor (TNF)-related apoptosis-inducing ligand (TRAIL), a member of the TNF superfamily, could induce apoptosis in various tumor cells and virus-infected cells, but not most normal cells [9]. It is recently reported that TRAIL induces apoptosis in HIV-infected macrophage [10], [11], but the exact underling mechanism is not well defined. TRAIL-induced apoptotic signaling pathway can be modulated by many factors. It is known that there are five TRAIL receptors, i.e., TRAIL receptor 1 (DR4), TRAIL receptor 2 (DR5/TRICK2/KILLER), TRAIL receptor 3 (decoy receptor 1, DcR1/TRID/LIT), TRAIL receptor 4 (decoy receptor 2, DcR2/TRUNDD) and osteoprotegerin (OPG) [12], [13]. There is a death domain in the intracellular region of DR4 or DR5, which can recruit death-inducing signaling complex (DISC) upon TRAIL stimulation, therefore, activate down stream caspase cascade leading to cell death by apoptosis. There is no intact death domain in the intracellular region of DcR1 and DcR2, and OPG, a soluble receptor, so that they are unable to induce apoptosis, even though they could compete with DR4 or DR5 for binding with TRAIL [14] and over-expression of DcR1 and/or DcR2 blocks TRAIL-mediated apoptosis in some cell types [12], [15]. It is reported that cellular FLICE-inhibitory protein (c-FLIP) suppresses the transduction of the death signal at the receptor level by occupying caspase-8 binding site on FADD thus blocking TRAIL-induced death signals [16], [17], and expression of inhibitor of apoptosis proteins including XIAP, c-IAP1, c-IAP2 and survivin suppresses activation of caspase cascade, therefore, protects the cells from apoptosis [18], [19], [20]. Bcl-2 family members, nuclear factor-kappa B (NF-κB) as well as PI3K/AKT could also affect TRAIL-induced apoptosis [17], [21], [22].

Herein, we established an HIV-1 Env-pseudotyped virus (HIV-1 PV)-infected MDM cell model to explore the molecular mechanism and signaling pathway, by which HIV-infected MDM could be eliminated by recombinant soluble TRAIL (rsTRAIL). Furthermore, we developed a more efficient method to eliminate the HIV-infected macrophage by combination of rsTRAIL with an agonistic anti-DR5 monoclonal antibody, which shows strong tumoricidal activity both in vitro and in vivo by a caspase-dependent and -independent manner [23]. It is significantly for the eradication of latent HIV-1 infection and AIDS.

## Materials and Methods

### HIV-1 PV production and titration

Env-expressing plasmid (pTHRO.18) and HIV-1 backbone plasmid lacking Env (pSG3△Env) were kindly provided by Professor Yiming Shao, Center for Disease Control (CDC), Beijing, China. HEK 293T/17 cells (ATCC, Maryland, USA) were co-transfected with 20 µg of pSG3△Env and 10 µg of pTHRO.18 plasmid DNA in a 10 cm tissue culture dish by a calcium phosphate method as previously described [24] and the medium was replaced with 37°C pre-warmed fresh medium 16 hr post transfection and incubated for an additional 48 hr. The virus-containing culture medium was harvested and clarified by centrifugation at 2000 g for 5 min and filtered through a 0.45-micron filter. The Env-pseudotyped HIV-1 virus (HIV-1 PV) was purified by centrifugation at 80,000 g and 4°C for 2 hr and re-suspended in complete medium and stored at −80°C till use. The medium from HEK 293T/17 cells transfected with pSG3△Env only was used as mock infection control. Titration was performed in TZM-bl cell [25] (AIDS Research and Reference Reagent Program, NIH) using a standard TCID_50_ (50% tissue culture infective dose) assay and HIV-1 p24 ELISA kit (ZeptoMetrix Corporation, MA, USA) according to the manufacturer's instructions.

### Isolation and infection of primary monocyte

Peripheral blood mononuclear cells (PBMCs) were isolated from HIV- and HBV- seronegative peripheral blood (supplied by Red Cross Blood Service Centre, Beijing, China) by centrifugation at 500 g for 20 min at room temperature on Ficoll-Paque solution. Monocyte-enriched fraction was isolated from PBMCs by a commonly used plastic adherence method [26], [27] or purified by CD14-positive selection using anti-CD14 microbeads (Miltenyi Biotec, Bergisch Gladbach, Germany) with a Midi MACS separator unit according to the manufacturer's instructions. Briefly, PBMCs were suspended in serum-free RPMI 1640 medium (GIBCO, Carlsbad, CA) at 5×10^6^ cells/ml and enabled the monocyte adherence to the plastic by incubating at 37°C in a humidified 5% CO_2_ incubator for one hour. After vigorously washed for three times with serum-free RPMI 1640, the adherent cells at a concentration of 0.5–1×10^6^ cells/ml were maintained in RPMI 1640 supplemented with 10% heat-inactivated fetal bovine serum (GIBCO, Carlsbad, CA), penicillin (100 U/ml), streptomycin (100 µg/ml), and M-CSF (250 ng/ml; Peprotech, Rocky Hill, NJ). The culture medium was half-exchanged every 3 days. The purity of the primary monocytes was examined by CD14 staining and flow cytometry. The monocytes (purity >90%) were allowed to differentiate for 7 days, and then were infected with HIV-1 PV at a multiplicity of infection (MOI) of ∼3 in the existence of polybrene at 10 ng/ml for 4 hr. MDM from the same donor treated with polybrene at 10 ng/ml and mock infection with the supernatant of HEK 293T/17 cells transfected with SG3△Env only were used as control. All culture supernatants, reagents and medium were endotoxin free.

### Assessment of cell viability

MDM cells were plated in 96-well plate at a density of 2×10^4^ cells per well and infected with HIV-1 PV for 7 days. The MDM cells were switched into fresh medium and treated with various concentration of rsTRAIL and/or AD5-10 [23] for a time course. The cell viability was determined by using CellTiter 96 aqueous nonradioactive cell proliferation assay (MTS) according to the manufacturer's instructions (Promega, Wisconsin, USA).

### Immunofluorescence assay

MDM cells (DPI = 7) on cover slips were fixed in fixative solution (V/V = 1∶1, ethanol/acetone) at −20°C for 10 min. After washed twice with PBS, the cells were incubated with sheep antibody against HIV-1 p24 (1∶80 dilution, the antibody was obtained through the AIDS Research and Reference Reagent Program, Division of AIDS, NIAID, NIH, from Dr. Michael Phelan) [28] for 45 min at room temperature and followed by FITC-conjugated mouse anti-sheep IgG monoclonal antibody (1∶200 dilution) staining for 30 min at room temperature. Hoechst 33342 (Sigma-Aldrich) was used for nuclear staining for 5 min at room temperature. After twice washing, the cover slip was put onto slide with mounting medium then painting the edge with a rim of nail polish and observed by fluorescence microscopy (Nikon Eclipse TE2000-U, Nikon).

### Detection of TRAIL receptors by flow cytometry

MDM cells were cultured in 6-well plate at a density of 1∼2×10^6^/ml in the presence of 250 ng/ml M-CSF (Peprotech, Rocky Hill, NJ) and infected with HIV-1 PV for 7 days. The cells were collected and washed with PBS, then re-suspended in FACS buffer (2% FBS in PBS with 0.1% sodium azide) and incubated with TRAIL receptor antibody (antibodies to DR4 and DR5, eBioscience, San Diego, CA; antibodies to DcR1 and DcR2 and the isotype control IgG, R&D systems, Minneapolis) at 4°C for 1 hr. After washed twice with FACS buffer, the cells were fixed in 2% paraformaldehyde and followed by flow cytometry (FACScan, Becton Dickinson, Germany). Mean fluorescence intensity of each receptor was assessed on the gated live-cell population with CellQuest software.

### Lentivirus infection

The recombinant lentiviral vectors expressing DcR1, DcR2 or the intracellular domain-deleted DR5 (DR5-delta) were constructed from pWPXL (Addgene plasmid 12257) and designated as pWPXL-DcR1, pWPXL-DcR2, pWPXL-DR5-delta, respectively. The target gene expression was driven by EF-1 alpha promoter plus intron. The lentiviral expression vector was transfected into HEK293T/17 cells with psPAX2 (Addgene plasmid 12260) and pMD2.G (Addgene plasmid 12259) using the calcium phosphate method as described above. Freshly isolated monocytes were cultured for 5 days followed by infection with the recombinant lentivirus and HIV-1 PV or mock for 7 days. The cells were harvested and assessed for the cell viability and the effect of expression of DcR1, DcR2 and DR5-delta on rsTRAIL- or AD5-10-mediated cell death.

### Western blot analysis

MDM cells were washed twice with phosphate-buffered saline and lysed in the SDS-PAGE sample buffer (Roche, Basel). Protein concentration was determined with BCA Protein Assay kit (Pierce, Rockford). 30 µg or 60 µg of total proteins were boiled for 5–10 min and subjected to 12% SDS-PAGE. The proteins separated in the gel were subsequently electrotransferred onto a Polyvinylidene Fluoride membrane (PVDF, Amersham Biosciences, Sweden). The membrane was blocked with 5% nonfat dry milk or BSA in TBS-T buffer (20 mM Tris-HCl, pH = 7.4; 8 g/L NaCl; 0.1% Tween 20) for 1 hr at room temperature followed by incubating with primary antibody (1∶1000 dilution for the antibody against caspase-3, caspase-8, caspase-9 and c-FLIP, Cell Signaling Technology; 1∶100 for anti-Bcl-XL, 1∶200 for anti-Bax, -Bid and -Bak, 1∶800 for anti-Bcl-2, Santa Cruz Biotechnology; 1 µg/ml of anti-actin, Sigma) in TBS-T buffer containing 5% nonfat dry milk or BSA at 4°C overnight. The membrane was washed for three times with TBS-T to remove any unbound primary antibody then probed with horseradish peroxidase-conjugated secondary antibody at room temperature for one hr. After washing three times with TBS-T, the interesting protein was visualized by using ECL Plus Western blotting detection system according to the manufacturer's instructions (Amersham Biosciences, Sweden).

### Measurement of reactive oxygen species (ROS)

Reactive oxygen species (ROS) generated in MDM cells was measured by using a ROS detection kit according to the manufacturer's instructions (Biyuntian Co. Jiangsu, China). Briefly, the HIV-1 PV-infected MDM cells were incubated in serum-free medium containing 10 µM DCFH-DA (Biyuntian Co. Jiangsu, China) for 20 min at 37°C. After washed for three times with RPMI 1640, the cells were cultured in the complete medium and treated with 200 ng/ml rsTRAIL and/or 200 ng/ml AD5-10 for 15, 30, 60, 120 and 240 min. Then the cells were harvested and subjected to detection of DCF fluorescence by flow cytometry (FACScan, Becton Dickinson, Germany).

### Statistical analysis

Data were presented as mean values ± S.D. and statistical significant was assessed by one-way ANOVA with Tukey's Multiple Comparison Test using Graphpad Prism version 4 (GraphPad Software, San Diego CA). For comparison of the expression of TRAIL receptors and other intracellular proteins, a two-tailed, two samples Student *t* test was used for evaluating statistical significance. *P* values were considered to be statistically significant when less than 0.05. All data shown are representative of at least 3 independent experiments.

## Results

### HIV-1 PV effectively infects primary MDM

**Figure 1:**
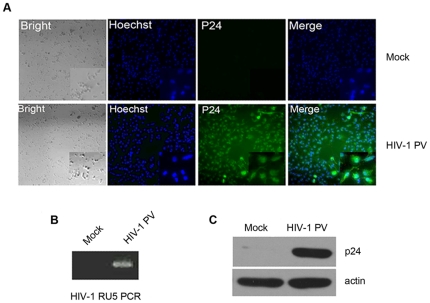
Infection of MDM by HIV-1 PV. Monocytes were isolated from the peripheral blood of healthy donors and culture for 7 days in RPMI 1640 supplemented with 10% heat-inactivated fetal bovine serum, penicillin (100 U/ml), streptomycin (100 µg/ml), and M-CSF (250 ng/ml) to allow the cell differentiation. The MDM cells were infected with HIV-1 PV at a multiplicity of infection (MOI) of ∼3 in the presence of 10 ng/ml of polybrene for 4 hr and cultured for 7 more days. MDM from the same donor treated with polybrene at 10 ng/ml and mock infected with the supernatant of HEK 293T/17 cells transfected with SG3ΔEnv only were used as control. The cells were analyzed with immunofluorescence staining (**A**) and Western blotting (**C**) for the viral p24 protein expression, and by HIV DNA PCR (**B**) for the presence of integrated proviral DNA.

Human monocytes were isolated and purified from healthy donors by Ficoll-Paque centrifugation and plastic adherence or by magnetic anti-CD14 microbeads. The primary monocytes (purity >90%) were allowed to be differentiated into monocyte-derived macrophage (MDM) and then infected with HIV-1 PV. The efficacy assay of HIV-1 PV infection by inverted fluorescence microscopy with FITC-labeled anti-HIV-1 p24 antibody demonstrated that more than 90% MDM cells were infected by the virus, but not the mock-infected control (Fig. 1A), indicating that uniform infection of the MDM cells by HIV-1 PV implemented. This result is consistent with previous report by Tsang et al. that MDM cells could well be infected by R5 tropic HIV-1 virus [29]. To confirm this observation, PCR by using HIV-1 PV-infected MDM DNA as template and HIV LTR-specific primers [30] was performed. It was shown that HIV-1 PV DNA was existed in the HIV-1 PV-infected MDM cells, but not in the non-infected cells (Fig. 1B). Similarly, HIV-1 p24 protein examined by Western blot assay was presented in the virus-infected MDM cells, but not in the mock-infected cells (Fig. 1C). These data confirm that HIV-1 PV infects human primary MDM cells effectively.

### Recombinant soluble TRAIL and anti-DR5 monoclonal antibody AD5-10 synergetically induces apoptosis in HIV-1 PV-infected MDM cells

**Figure 2:**
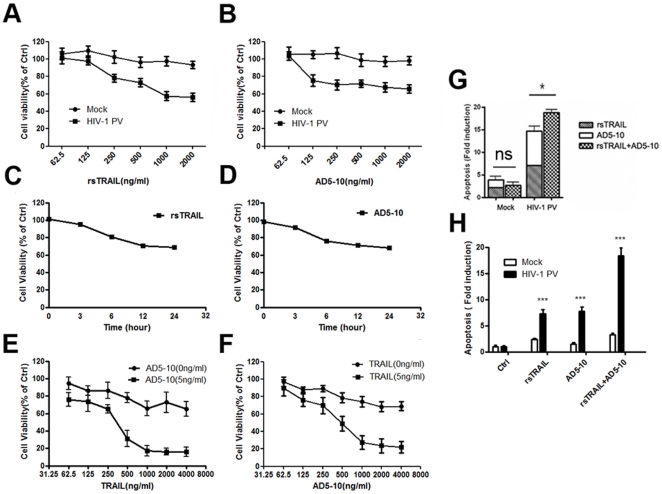
rsTRAIL and AD5-10 synergetically induces apoptosis in HIV-1 PV-infected MDM. HIV-1 PV-infected MDM cells were treated with increasing concentration of rsTRAIL (**A**) or AD5-10 (**B**). The cell viability was determined by MTS assay. **C and D**, the virus-infected MDM cells were treated either with 500 ng/ml of rsTRAIL or 500 ng/ml of AD5-10 for 3, 6, 12, and 24 hr. The virus-infected MDM cells were overnight incubated with or without 5 ng/ml of AD5-10 and rsTRAIL at indicated concentration (**E**), or 5 ng/ml of rsTRAIL and AD5-10 at indicated concentration (**F**). **G and H**, the virus-infected MDM cells were incubated with 500 ng/ml rsTRAIL and/or 500 ng/ml AD5-10 for 6 hr, and the apoptotic nucleosome was detected by Cell Death Detection ELISA kit. Values are represented results of three independent experiments with error bar representing standard deviation of the mean. *, *p*<0.05; ***, *p*<0.001; *ns*, no significant.

To investigate whether recombinant soluble TRAIL (rsTRAIL) as well as agonistic anti-DR5 antibody, AD5-10, could induce HIV-1 PV-infected MDM cell death in the model system, HIV-1 PV-infected and mock-infected MDM cells were treated with rsTRAIL or/and AD5-10 at indicated concentration for 12 hours and followed by MTS assay for the cell viability. As shown in Fig. 2A–D, rsTRAIL and AD5-10 suppressed the viability of HIV-1 PV-infected MDM cells in a dose- and time-dependent manner. The combination of rsTRAIL and AD5-10 more effectively inhibited the viability of HIV-1 PV-infected MDM cells (Fig. 2E and 2F). DNA fragmentation ELISA confirmed that rsTRAIL and AD5-10 significantly induced apoptosis of HIV-1 PV-infected MDM cells, but not the mock-infected cells (Fig. 2G and 2H), demonstrating that both rsTRAIL and AD5-10 could induce cell death of the HIV-1-infected MDM by apoptosis. Furthermore, rsTRAIL and AD5-10 synergetically induced apoptosis of the HIV-1-infected MDM cells.

### HIV-1 PV infection suppresses expression of TRAIL decoy receptors and c-FLIP

**Figure 3:**
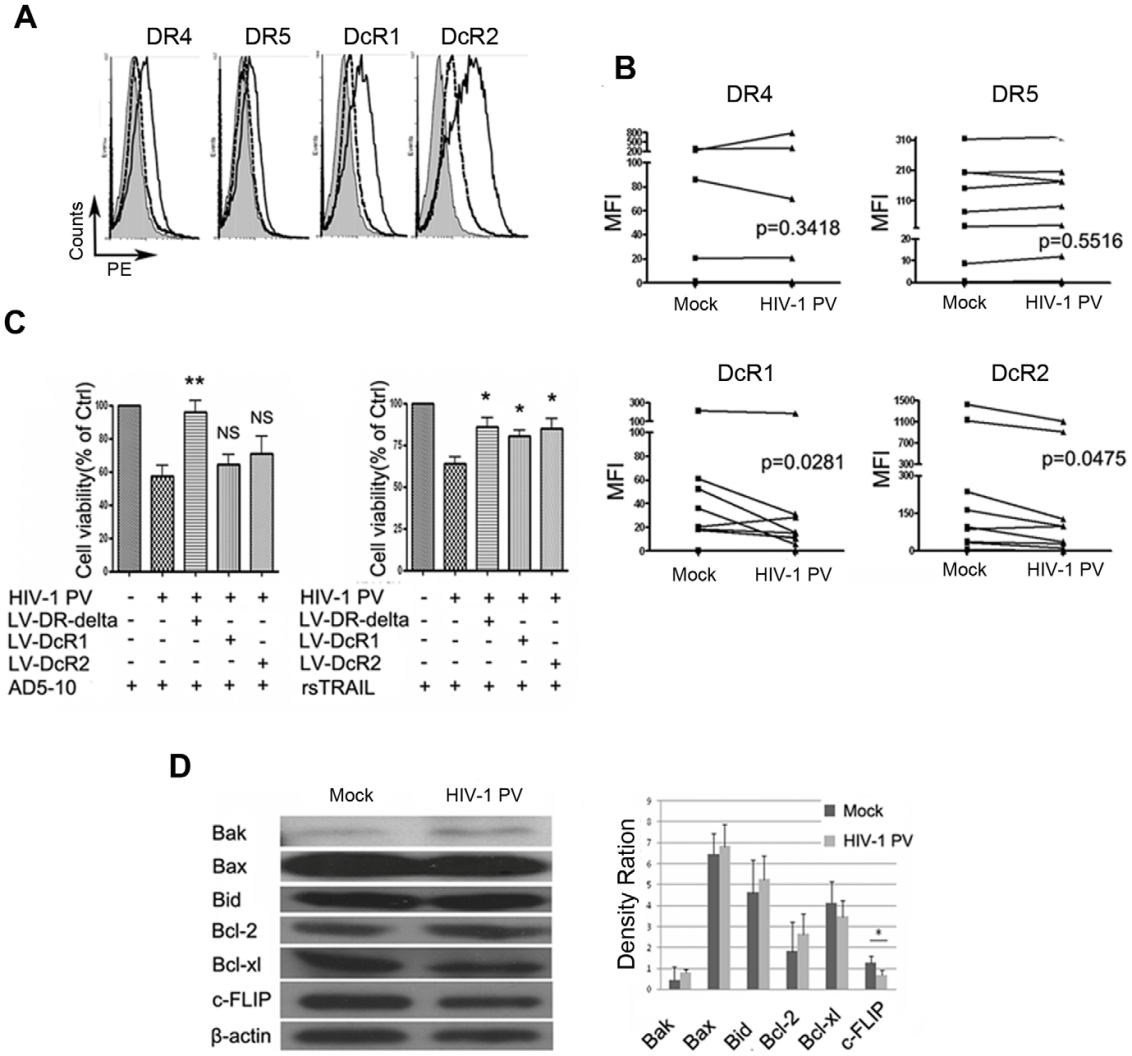
HIV-1 PV infection suppresses expression of TRAIL decoy receptors and c-FLIP. The virus-infected MDM were collected and stained with PE-labeled anti- DR4, -DR5, -DcR1, -DcR2 antibody and the isotype control, respectively, and followed by flow cytometry. **A**, representative analysis of TRAIL receptor expression on mock- or virus-infected MDM, Open histograms with solid lines, mock-infected MDM stained with PE-labeled anti-TRAIL receptor mAbs; Open histograms with dashed lines, HIV-1 PV infected MDM stained with PE-labeled anti-TRAIL receptor mAbs; shaded histograms, mock-infected MDM stained with PE-labeled isotype control. **B**, statistical results from 12 samples. The MFI (mean fluorescent intensities) of TRAIL receptor was assessed by flow cytometry and analyzed by a two tailed, two sample student *t* test (*P* values were indicated). **C**, lentivirus-mediated over-expression of DR5-delta, DcR1 and DcR2 on MDM rescued rsTRAIL- or AD5-10-induced cell death. MDM cells were infected with lentivirus vectors expressing DR5-delta, DcR1 or DcR2, respectively. HIV-1 PV-infected MDM cells were treated with 500 ng/ml of rsTRAIL or 500 ng/ml of AD5-10 for 12 hr. The cell viability was examined by MTS assay. Data are representative of three independent experiments. **D**, expressions of Bcl-2 family proteins and c-FLIP in HIV-1 PV-infected MDM were analyzed by Western blot assay. Data is presented as mean ± S.D. of 12 independent experiments. *, *p*<0.05; **, *p*<0.01 compared with mock or HIV-1 PV.

To explore the possible underlying mechanisms of rsTRAIL and/or AD5-10 induced apoptosis in the HIV-1 PV-infected MDM cells, expression of TRAIL receptors on the virus infected MDM cells were examined by flow cytometry after staining with PE-labeled specific antibodies. As shown in Fig. 3A and 3B, TRAIL decoy receptor, DcR1 and DcR2 expression in MDM cells infected with HIV-1 PV were significantly down-regulated on the 7th day post infection compared with mock-infected control (n = 12; p = 0.0281 for DcR1, p = 0.0475 for DcR2). Interestingly, DR4 and DR5 expression was not affected by the virus infection compared with mock control (n = 12; p = 0.3418 for DR4, p = 0.5516 for DR5). To demonstrate whether the decoy receptor over-expression affects TRAIL- or AD5-10-induced apoptosis of HIV-1 PV-infected MDM cells, MDM cells were transformed with a lentivirus vector encoding DcR1, DcR2 or dominant negative DR5 (intracellular domain deleted DR5, DR5-delta) followed by HIV-1 PV infection and treatment with rsTRAIL or AD5-10. The cell viability was tested by MTS assay followed by flow cytometry. As shown in Fig. 3C, dominant negative DR5 expression well rescued rsTRAIL- or AD5-10-induced cell death in HIV-1 PV-infected MDM cells. In contrast, the over-expression of DcR1 or DcR2 rescued rsTRAIL- but not AD5-10-induced cell death. These data reveal that down-regulation of TRAIL decoy receptor facilitates TRAIL-induced cell death in HIV-1 PV-infected MDM cells.

It is well known that some intracellular proteins could regulate TRAIL receptor-mediated signaling pathway and cell death, such as c-FLIP and Bcl-2 family members. We therefore further examined the expression of c-FLIP and Bcl-2 family members by Western blot analysis in HIV-1 PV- and mock-infected MDM cells. As shown in Fig. 3D, HIV-1 PV-infection down-regulated c-FLIP expression in the cells but did not affect the expression of Bcl-2 family members tested, suggesting that down-regulated TRAIL decoy receptors and c-FLIP expression facilitate TRAIL-induced apoptosis in HIV-1 PV-infected MDM.

### Combination of rsTRAIL and AD5-10 induces a caspase-dependent and -independent cell death pathway

**Figure 4:**
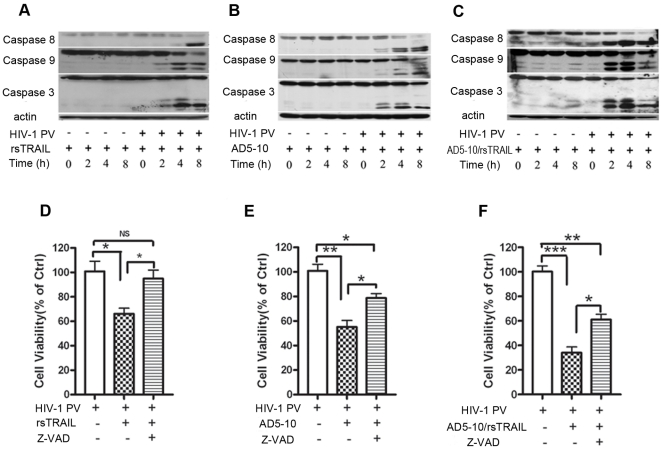
RsTRAIL and AD5-10 induced caspase-dependent and -independent cell death. **A-C**, Western blot analysis of caspase activation. HIV-1 PV infected MDM cells were treated with either 250 ng/ml rsTRAIL (A) or 250 ng/ml AD5-10 (B) or 250 ng/ml rsTRAIL plus 250 ng/ml AD5-10 (C) for 2, 4, 6 and 8 hr. The cells were lysed and the lysates were subjected to SDS-PAGE and immunoblotting with anti-caspase -3, -8, or -9 antibody. **D**–**E**, cell viability determined by MTS. HIV-1 PV infected MDM were treated with 500 ng/ml rsTRAIL (D) or 500 ng/ml AD5-10 (E) or 250 ng/ml rsTRAIL plus 250 ng/ml AD5-10 (F) in the absence or presence of 50 µM of Z-VAD-fmk for 12 hr. Values are mean of three independent experiments with error bar representing standard deviation of the mean. *, *p*<0.05; **, *p*<0.01; ***, *p*<0.001; *ns*, no significant.

Next, we investigated TRAIL or/and AD5-10-induced signaling pathway in HIV-1 PV -infected MDM cells. HIV-1 PV-infected MDM cells were treated with rsTRAIL and/or AD5-10 for the indicated time followed by Western blot assay with the specific antibodies against caspase-8, -9, -3 to examine caspase activation. As shown in Fig. 4A–C, rsTRAIL or/and AD5-10 treatment resulted in the cleavage of caspase-8, -9, and -3, indicating that rsTRAIL or/and AD5-10 trigger the caspase activation in HIV-1 PV-infected MDM cells, but not in the mock-infected cells. However, while a pan-caspase inhibitor, Z-VAD fmk, was used to block caspase-dependent cell death in the cells, TRAIL-induced apoptosis was well blocked, but the cell death induced by AD5-10 as well as the combination of AD5-10 plus rsTRAIL was only partially inhibited in the cells (Fig. 4D-F), suggesting that the combination of rsTRAIL and AD5-10 induces cell death in the HIV-1 PV-infected MDM cells through a caspase-dependent and -independent signaling pathway.

### ROS generation and JNK phosphorylation are involved in the cell death of HIV-1 PV-infected MDM cells

**Figure 5:**
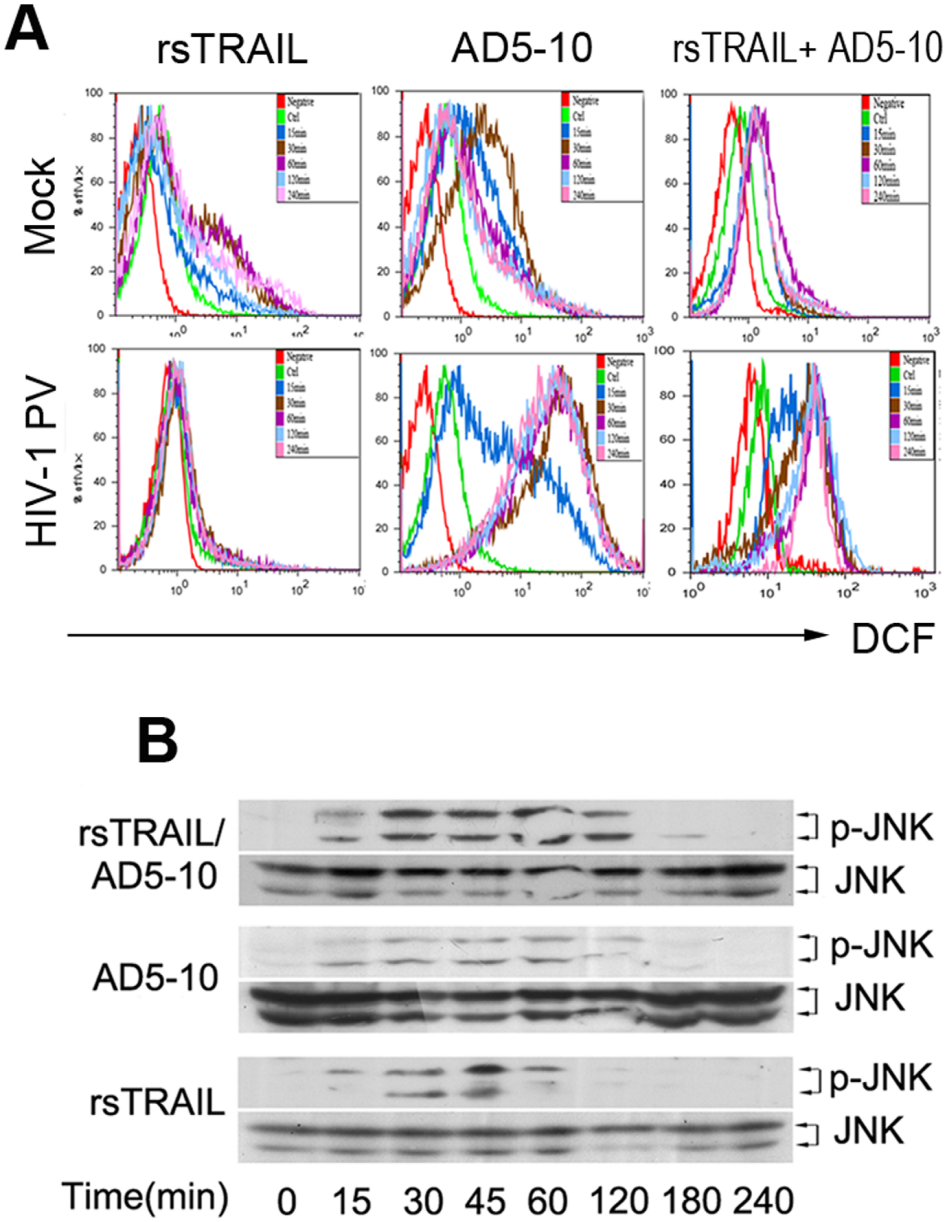
ROS generation and JNK phosphorylation involves in AD5-10 induced cell death. **A**, HIV-1 PV- or mock-infected MDM cells were treated with 200 ng/ml rsTRAIL (left) or 200 ng/ml AD5-10 (middle) or 200 ng/ml rsTRAIL plus 200 ng/ml AD5-10 (right) for 15, 30, 60, 120, 240 min. ROS generation was examined by loading with DCFH and followed by flow cytometry. **B**, JNK phosphorylation was examined with indicated specific antibody (1∶1000 dilution, phosphor-SAPK/JNK(Thr183/Tyr185) and JNK antibody) in HIV-1 PV-infected MDM cells (DPI = 7) treated with 500 ng/ml rsTRAIL (bottom) or 500 ng/ml AD5-10 (middle) or 250 ng/ml rsTRAIL plus 250 ng/ml (top) for indicated time. Data was obtained from three independent assays and a typical experiment is presented.

To explore the signaling molecules in the combination of rsTRAIL and AD5-10 induced caspase-independent pathway, we further investigated whether ROS generation was related to the cell death of HIV-1 PV-infected MDM cells. As shown in Fig. 5A, AD5-10, but not rsTRAIL, induced ROS generation in the HIV-1 PV-infected MDM cells. Since ROS could contribute to the phosphorylation of JNK, which is an important event in the cell death signaling, we further tested phosphorylation of JNK in HIV-1 PV-infected MDM cells treated with rsTRAIL or/and AD5-10. As shown in Fig. 5B, Western blot assay demonstrated that JNK phosphorylation occurred affirmatively. These data indicate that ROS generation and JNK phosphorylation are involved in the cell death of the HIV-1 PV-infected MDM cells.

## Discussion

In the present study, we have established HIV-1 PV-infected MDM as a model system to study the effect of HIV-1 infection on the cell apoptosis and signaling pathway. We firstly demonstrated that HIV-1-infected MDM cells were more sensitive to the recombinant soluble TRAIL (rsTRAIL) and the agonistic anti-DR5 monoclonal antibody AD5-10 induced cell death. The receptor expression study showed that TRAIL decoy receptors DcR1 and DcR2 were down-regulated, but DR4 and DR5 expression remained no changes, suggesting that DcR1 and DcR2 expression disregulation might contribute to the susceptibility of HIV-1 infected MDM cells to TRAIL and/or AD5-10 induced cell death. To confirm this hypothesis, we further demonstrated that lentivirus mediated over-expression of dominant negative DR5 (DR5-delta) well rescued rsTRAIL- or AD5-10-induced cell death in HIV-1 PV-infected MDM cells, but DcR1 or DcR2 over-expression only rescued rsTRAIL- but not AD5-10-induced cell death since AD5-10 is specifically to DR5 but not to DcR1 or DcR2 [23]. However, this finding does not consist with the report by Lum, et al. that HIV infection up-regulates DR5, but neither DcR1 nor DcR2 expression in MDM cells of HIV positive subjects [11]. As a matter of fact, dissymmetry expression of TRAIL receptors are often observed in virus-infected cells as well as various tumor cells, which are believed to account for the susceptibility or resistance to TRAIL-induced apoptosis [31], [32], [33]. Down-regulation of the DcRs in cancer renders cancer cells more susceptible to TRAIL-induced apoptosis, which could be counted as a protective response against tumor formation or progression. It is reported that hypermethylation of the promoters of DcR1 and DcR2 is important in the down-regulation of DcR1 and DcR2 expression in some tumor types [34]. Methylation of DcR1 and DcR2 gene may attribute to a secondary effect of the methylation of DR4 and DR5, meanwhile, methylation of the two decoy receptors may occurred independently of each other and of DR4 and DR5. Down-regulation of DcR1 and DcR2 in HIV-1 infected MDM may contribute to epigenetic regulation in a DR4 and DR5-independent manner. Consequently, down-regulation of the decoy receptors weakens the cell competing capacity with TRAIL and strengthens the cell binding activity to TRAIL through DR5, therefore, results in more cell death in the HIV-1 PV-infected MDM cells.

However, it is known that expression levels of TRAIL receptors do not always correlate with the cell sensitivity to TRAIL cytotoxicity. A number of intracellular molecules play important roles in the regulation of apoptotic signaling, such as c-FLIP [16], [35], [36], IAPs (inhibitor of apoptosis proteins) [37] and Bcl-2 family proteins [38]. Examination by Western blotting showed that c-FLIP expression was down-regulated, but without notable exchanges in Bcl-2 family member expression in the HIV-1 pseudotyped virus-infected MDM cells compared with mock control. It was reported in the literature that Bcl-2 expression decreased transiently and followed by restoration to the initial level in lymphoblastoid T (J.Jhan) or monocytic (U937) cells [39]. This discrimination may caused by different cells used in the experiments. c-FLIP is a catalytically inactive caspase-8/10 homologue, which interferes with DISC (death inducing signal complex) formation in the extrinsic cell death pathway, therefore, serves as a key inhibitor of death receptor-induced apoptosis. In addition, c-FLIP could activate NF-κB by recruiting tumor necrosis factor receptor-associated factor 2 (TRAF-2) and receptor-interacting protein-1 (RIP1) into DISC, therefore, promote cell survival and proliferation [40], [41]. However, at low concentration, c-FLIP heterodimerizes with procaspase-8 and induces caspase-8 autoprocessing as well as apoptosis [42]. In the present study we observed that down regulation of c-FLIP expression was occurred in the HIV-1-infected MDM cells, but no changes in Bcl-2 family proteins, indicating that down regulation of c-FLIP expression facilitates death receptor-mediated cell death.

Western blotting analysis of caspase activation in the virus-infected MDM cells showed that TRAIL or/and AD5-10 treatment activated caspase-8, -9, and -3 in HIV-1 infected MDM cells, indicating that TRAIL or/and AD5-10 trigger the caspase cascade. While a pan-caspase inhibitor, Z-VAD fmk, was used, TRAIL-induced apoptosis was well blocked, but the cell death induced by AD5-10 as well as the combination of AD5-10 plus rsTRAIL was only partially inhibited, indicating that caspase-dependent apoptosis is activated by TRAIL- or TRAIL plus AD5-10-induced cell death, but AD5-10 itself could also induce caspase-independent cell death in the HIV-1 PV-infected MDM cells.

Finally, we demonstrated that ROS generation and JNK phosphorylation were involved in TRAIL-mediated apoptosis of HIV-1-infected MDM cells. ROS is known to be involved in the early stage of apoptosis, and induces the depolarization of the mitochondrial membrane [43]. We showed that cross link of DR5 by the anti-DR5 mAb, AD5-10, induced production of ROS and subsequent apoptosis in HIV-1 infected MDM. Meanwhile, JNK phosphorylation was detected along with ROS generation. It is known that JNK participates in the apoptotic signaling pathway initiated by stress or toxic stimuli [44]. These data suggest that ROS generation in abundance in HIV-1 infected MDM upon AD5-10 stimulation may further activate ROS-JNK-NF-κB pathway and induces cell death by apoptosis as we have reported in tumor cell model [45].

In summary, we have demonstrated that HIV infection facilitates TRAIL-induced cell death in monocyte-derived macrophage by down regulating the expression of TRAIL decoy receptors and intracellular c-FLIP. Meanwhile, the agonistic anti-DR5 antibody, AD5-10, induces apoptosis synergistically with TRAIL in HIV-1-infected cells. ROS generation and JNK phosphorylation are involved in this process. These findings potentiate clinical usage of the combination of TRAIL and AD5-10 for eradication of HIV-infected macrophage as well as AIDS.
